# A Modified Fixation Method for Talonavicular Arthrodesis in the Treatment of Müller‐Weiss Disease: The Use of the Shape‐Memory Alloy Staple as an Adjunct

**DOI:** 10.1111/os.14205

**Published:** 2024-08-27

**Authors:** Zhao‐Ying Lv, Yuan‐Hao Tong, Bai‐Hui Wu, Yu‐Zhong Zheng, Xiao‐Yong Lin, Yi‐Qiu Lin, Chen Xiao Zheng

**Affiliations:** ^1^ Zhongshan Hospital of Traditional Chinese Medicine Affiliated to Guangzhou University of Traditional Chinese Medicine Zhongshan China

**Keywords:** Müller‐Weiss Disease, TalonavicularArthrodesis, The Shape‐Memory Alloys Staple

## Abstract

**Objective:**

Arthrodesis, usage of metallic implants for internal fixation, is commonly employed as the primary treatment modality for Müller‐Weiss disease (MWD). Nevertheless, the efficacy of the current methods of fixation leaves room for improvement. Inadequate fixation strength and the risk of fixation failure are both critical concerns requiring attention. This study explored the clinical effects of implementing a modified fixation technique in talonavicular arthrodesis for the treatment of MWD.

**Methods:**

A total of 14 cases diagnosed with MWD undergoing talonavicular (TN) arthrodesis from January 2021 toMarch 2023 were included in the retrospective study. The fixation method for fusion involved the use of screws, with additional support from the shape‐memory alloy (SMA) staple. Relevant clinical outcomes and complications were evaluated preoperatively and postoperatively. Paired‐samples *t*‐test was used for all data comparisons.

**Results:**

Radiographic evidence confirmed solid fusion, and follow‐up evaluations showed satisfactory results in all cases. The American Orthopedic Foot and Ankle Society (AOFAS) scores were elevated from 32.21 ± 4.0 (range: 22–38) preoperatively to 86.5 ± 2.7 (range: 81–90) postoperatively (*p* < 0.001). The visual analog scale (VAS) scores declined from 7.40 ± 0.8 (range: 6–8.5) preoperatively to 1.21 ± 1.1 (range: 0–3) postoperatively (*p* < 0.001). The lateral Meary's angle changed from 13.50 ± 5.2 (range: 8–24) preoperatively to 4.14 ± 2.9 (range: 1–11) degrees postoperatively (*p* < 0.001). The calcaneal pitch angle increased from 10.07 ± 4.0 (range: 5–19) preoperatively to 14.35 ± 4.0 (range: 8–21) degrees postoperatively (*p* < 0.001). The talar‐first metatarsal angle decreased from 11.71 ± 3.8 (range: 8–18) preoperatively to 4.28 ± 3.1 (range: 0–9) degrees postoperatively (*p* < 0.001). One patient was observed to experience delayed wound healing and wound infection. No nerve damage, malunion, pseudoarthrosis, or fixation failure were observed.

**Conclusion:**

The results indicated that the fusion of the TN joint using a combination of screws and shape memory alloy staples, could lead to favorable clinical outcomes and significantly enhance the quality of life for patients with MWD. This technique is not only safe and effective but also straightforward to perform.

## Graphical abstract

Using the SMA staple as an adjunct alongside screws in talonavicular arthrodesis surgery to treat MWD provides good clinical outcomes. We took the method of putting the SMA staple on the basis of the existing screws. The study emphasizes the importance of a better place for orthopedic implants to assist bone healing, and the efficacy of the SMA staple in clinical applications was further evaluated. Therefore, for surgical interventions targeting MWD, this modified fixation method presents a promising option.

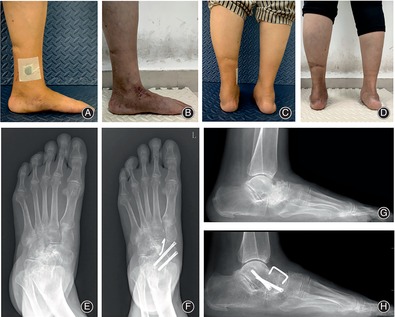

## Introduction

MWD is an uncommon foot disease characterized by sclerosis, deformity, and fragmentation of the navicular, resulting in perinavicular osteoarthritis.^1^ Patients often suffer chronic and progressive midfoot and hindfoot pain, and the incidence in women is higher than that in men. Maceira^2^ presented a staging system for MWD which is now commonly used for assessing the progress of the condition. The pathological changes show different degrees of the compression of the navicular, the dorsal dislocation of the navicular on the talar head, and the lowering of the height of the longitudinal arch. The TN‐cuneiform (TNC) joint will be formed when the navicular bone is completely extruded.^3^

Patients tend to try conservative treatment such as nonsteroidal anti‐inflammatory drugs to relieve pain, or insole with medial longitudinal arch support. The factors associated with failure of conservative treatment contain midfoot abduction and radiographic TN arthritis, and so on.^4^ When the disease reaches stage III or the prolonged conservative treatment fails, surgery may be indicated.

At present, no optimal surgical treatment exists for MWD. Arthrodesis, including TN arthrodesis, TNC arthrodesis, or triple arthrodesis, remains the mainstay treatment for MWD.^5^, ^6^, ^7^, ^8^ The fundamental concept of arthrodesis involves the restoration of the injured joint surface, achieved by its removal, and the subsequent immobilization of the adjacent bone to form a stable structure. The execution of the fusion process necessitates the use of thread hollow screws, or a locking plate for secure anchorage.

However, we encountered some problems in clinical practice. First, intraoperative restoration of talus rotation was the key to normal alignment of the subtalar joint/TN joint, nonetheless, the capacity of the screw to resist talus rotation is constrained. Screw fixation might be inadequate to counteract the residual forces arising from the external rotation of the talar head and the adduction forces acting on the navicular, respectively.^9^ Second, during the surgical procedure, it was observed that the act of drilling in screws can exacerbate bone fragmentation, potentially compromising the optimal strength of fixation. Third, we found that loosening or breakage of the internal fixation material may occur during postoperative follow‐up, necessitating a second operation.

The SMA staple belongs to a metallic alloy composed of nickel and titanium, featuring super‐elasticity and shape memory characteristics. This kind of alloy material can recover its original shape before low‐temperature deformation after being heated, which has been successfully used in osteotomies, arthrodesis, and fracture fixation, particularly in small bones. Possessing inherent compressive properties contributes to the creation of a stable fracture environment.^10^

The objectives of this retrospective study included: (i) Investigating the clinical and radiographic outcomes of the TN arthrodesis when using the SMA staple as an adjunct. (ii) Observing whether the combination of screws and the SMA staple achieved effective fusion of the joint, and whether any complications related to failure of any internal fixation occurred. (iii) Further confirming that TN arthrodesis could correct the force line of the foot and significantly improve the symptoms of patients with MWD.

## Materials and Methods

### Inclusion and Exclusion Criteria

Patients were included if: (1) A complaint of midfoot pain persisted for over 5 months, and failed to respond to conservative treatment exceeding 2 months. (2) The patient was confirmed (by MRI or CT scan) as having MWD. (3) The surgical method was TN arthrodesis, and the SMA staples were used as an adjunct.

Patients were excluded if: (1) Hypertension, diabetes, or peripheral vascular disease were uncontrolled. (2) Underwent previous traumatic or stress fractures of the navicular. (3) If rheumatoid arthritis or osteoporosis was present. (4) Faced with loss of follow‐up.

A total of 14 patients were reviewed in this study from January 2021 to March 2023, retrospectively. All patients underwent anteroposterior (AP) and lateral weight‐bearing radiographs of the foot preoperatively and postoperatively. The CT and/or MRI examinations were applied in all patients preoperatively. All patients underwent successful surgical treatment, and there was no attrition to surgical follow‐up. This study has been approved by the Medical Ethics Committee (Code Number 2024ZSZY‐LLK‐374), and informed consent was obtained from all included patients.

### Method of Operation

#### Anesthesia and Position

The procedure was performed under successful spinal anesthesia. Patients were in a supine position. A thigh tourniquet was used. The surgical site was disinfected and draped as usual.

#### Joint Debridement and Reduction

The TN joint was exposed through a dorsal‐medial approach lateral to the anterior tibial tendon. The talus and the navicular bone were released and reduced to the fullest extent feasible. The articular surface and the fragmented navicular bone pieces were removed by bone rongeur and osteotome using a freehand maneuver. External rotation of the talar head was corrected until the TN joint was restored, midfoot abduction and the force line were corrected. The positon was maintained by the Kirschner wire temporarily and confirmed satisfactorily by intraoperative fluoroscopy.

#### Fixation

**FIGURE 1:**
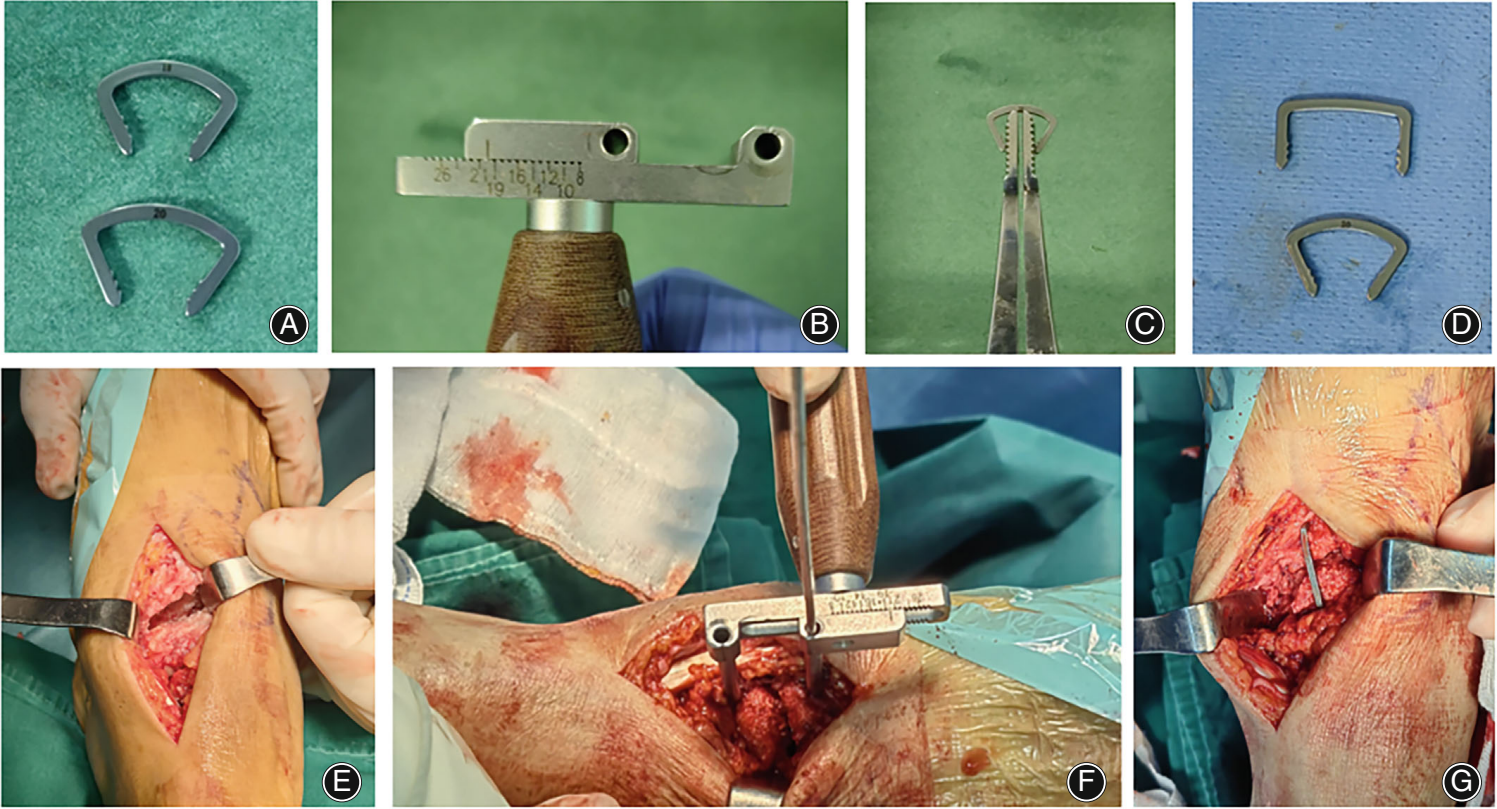
(A) Models: 18and 20mm; (B) locator: adjusting the drilling depth; (C) molder: spreading the shape‐memory alloy (SMA) staples; (D) comparison of the SMA staple before and after molding; (E) debride the articular surfaces to the subchondral bone; (F) use the locator for drilling holes on the bone; (G) completion of a SMA staple fixation.

Fixation of the talus‐navicular bone used two hollow screws and the SMA staple (Shanghai Xin Chang Memory Alloy Tech Co., Ltd.) placed perpendicular to the articular surface. The SMA staple should be pre‐cooled in ice water to make shaping easier. We used a spreader to adjust the nails to the desired length and promptly implanted them into pre‐drilled holes. Tapping the two legs of the staple ensured that the back of the staple was tightly fixed against the periosteal surface. Any significant delay may result in the SMA staple shortening (Figure 1). As determined by the individual situation, the iliac cancellous bone graft of appropriate size was harvested from the ipsilateral iliac crest and filled at the gap of the arthrodesis site. Then we irrigated the area, sutured it in layers, and dressed the wound.

#### Postoperative Management

A plaster cast was applied for 6 weeks for external protection. The patient underwent radiological examinations at 1, 2, and 3 months postoperatively. Full weight‐bearing was started after clinical and radiographic union had been documented.

### Evaluation Indicators

**FIGURE 2:**
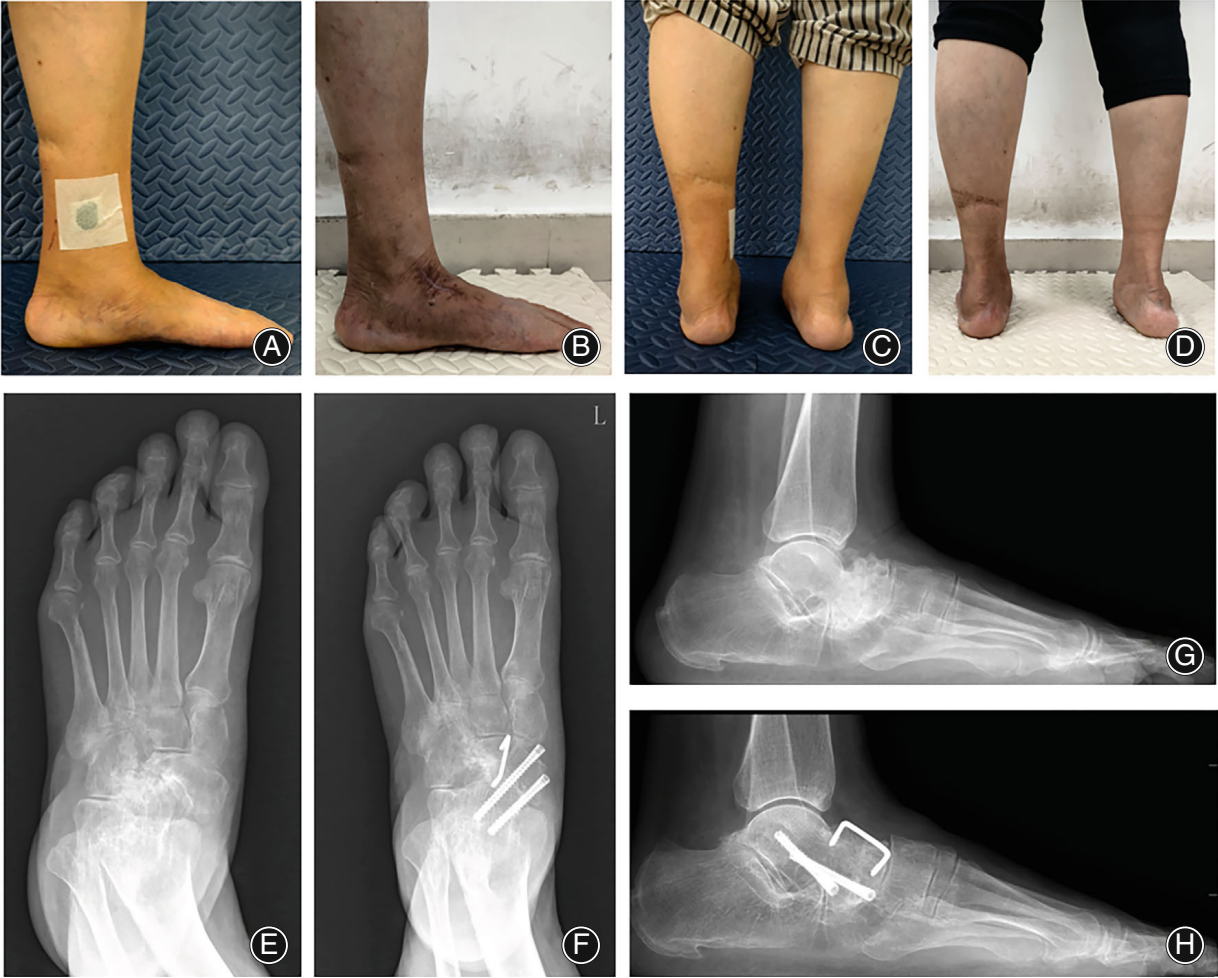
(A,C) preoperative appearance image; (B,D) postoperative appearance image; (E,G) the lateral and anteroposterior (AP) weight‐bearing radiographs of foot preoperatively; (F,H) the lateral and AP weight‐bearing radiographs of foot postoperatively. The force line was corrected.

The radiographic parameters (including the Calcaneal pitch angle, the lateral Meary's angle, the talar‐first metatarsal angle) on the lateral and AP weight‐bearing radiographs of the foot were measured preoperatively and 3 months after the surgery (Figure 2). The union time was determined by the follow‐up radiographs. The fusion was considered successful when the bone trabeculae lines were across the fusion site. Complications were recorded exactly during and after surgery.

The ankle‐hindfoot scale of the AOFAS midfoot scale and the VAS were used to evaluate patients before surgery and at the final interview.The follow‐up was conducted every 1month within 3months after operation, and 3–6 months thereafter.

### Statistical Methods

The paired *t* test was performed to evaluate the radiographic parameter and clinical functional outcomes. All data were analyzed using SPSS 25.0 (IBM, Chicago) statistical software. Data were expressed as mean ± SD. The *α* value of the inspection level was set to 0.05.

## Results

### Patient Information

A total of 14 patients (3 men and 11 women) diagnosed with MWD were enrolled in our research. The average age when the patients underwent operation was 58.6 ± 6.6 (range: 51–72) years old. According to the Maceira classifications, there were 8 stage III and 6 stage IV patients. The duration of the pain symptoms was 4.6 ± 4.0 years (range: 0.5–10 years). The operative side contains 6 left and 8 right sides. The follow‐up duration ranged from 12 to 38 months with a mean of 24.1 ± 7.9 months. The average body mass index was 25.8 ± 2.7 kg/m^2^ (Table 1).

**TABLE 1:** Basic information of the included 14 patients.

Age (years)	58.6 ± 6.6
Gender (male/female)	3/11
Maceira stage (III/IV)	8/6
Symptom duration (years)	4.6 ± 4.0
Operative site (left/right)	6/8
Follow‐up duration (months)	24.1 ± 7.9
BMI (kg/m^2^)	25.8 ± 2.7

*Note*: Data are presented as means ± standard deviations.

Abbreviation: BMI, body mass index.

### Clinical Results

At the last follow‐up, the AOFAS scores were elevated from 32.21 ± 4.0 (range: 22–38) preoperatively to 86.5 ± 2.7 (range: 81–90) postoperatively (*p* < 0.001). The VAS scores reduced from 7.40 ± 0.8 (range: 6–8.5) preoperatively to 1.21 ± 1.1 (range: 0–3) postoperatively (*p* < 0.001).

The lateral Meary's angle changed from 13.50 ± 5.2 (range: 8–24) preoperatively to 4.14 ± 2.9 (range: 1–11) degrees postoperatively (*p* < 0.001). The Calcaneal pitch angle increased from 10.07 ± 4.0 (range: 5–19) preoperatively to 14.35 ± 4.0 (range: 8–21) degrees postoperatively (*p* < 0.001). The talar‐first metatarsal angle decreased from 11.71 ± 3.8 (range: 8–18) preoperatively to 4.28 ± 3.1 (range: 0–9) degrees postoperatively (*p* < 0.001). At the end of the follow‐up visit, a 100% union was achieved. The average time when patients achieved bone fusion after the surgery was 3.39 ± 0.4 (range: 3–4) months (Table 2).

**TABLE 2:** Comparison of preoperative and postoperative radiographic results and functional scores.

	Preoperative	95% CI	Postoperative	95% CI	*p*‐value
VAS	7.40 ± 0.8	6.92,7.86	1.21 ± 1.1	0.61,1.82	<0.001
AOFAS	32.21 ± 4.0	29.90,34.52	86.57 ± 2.7	85.00,88.14	<0.001
The lateral Meary's angle (°)	13.50 ± 5.2	10.49,16.51	4.14 ± 2.9	2.48,5.80	<0.001
The calcaneal pitch angle (°)	10.07 ± 4.0	7.75,12.40	14.35 ± 4.0	12.09,16.63	<0.001
The talar‐first metatarsal angle (°)	11.71 ± 3.8	9.51,13.91	4.28 ± 3.1	2.50,6.08	<0.001
Bone fusion (month)			3.39 ± 0.4		

*Note*: Data are presented as means ± standard deviations.

Abbreviations: CI, confidence interval; VAS, visual analogue score.

### Complication

One patient was observed to suffer from delayed wound healing, and then developed wound infection at postoperative 7 weeks, which resolved after debridement and vacuum sealing drainage, then had an uneventful follow‐up period. During follow‐up, none of the other patients experienced any complications such as infection, delayed wound healing, nerve damage, malunion, pseudoarthrosis or fixation failure including loosening, breakage, or displacement. All the patients were satisfied with the outcomes of the surgery at their last follow‐up visit (Table 3).

**TABLE 3:** Postoperative complications.

Delayed wound healing	1
Infection	1
Implant displacement	0
Implant loosening	0
Implant breakage	0
Nerve damage	0
Deformity	0
Recurrence	0
Pseudoarthrosis	0

*Note*: Number: the number of each complication.

## Discussion

The main objective of this study was to observe efficacy of this method of fixation in joint healing and incidence of complications associated with fixation failure. During the follow‐up period, significant improvements were observed in both pain level and foot function for the included patients. No fixation failures were observed. These findings hold implications in providing insights for the usage of SMA staples in optimizing the process of arthrodesis surgery.

### Current Fixation Methods

Currently, arthrodesis continues to be the primary treatment for managing MWD.^5^, ^6^, ^7^, ^8^ The aims of the TN arthrodesis include preventing the external rotation of the talar head, correcting the varus of the hindfoot, and recovering the force line. When bone destruction affects adjacent joints, TN arthrodesis, TNC arthrodesis, or triple arthrodesis will be considered. Some scholars advocate for procedures such as calcaneal osteotomy^11^ to redistribute weight‐bearing in the foot, or TNC arthrodesis with fusion of a rectangular bed^12^ to treat MWD. To assess the condition of the tarsal bones and all adjacent joints, and to determine which joint should be fused during surgery. Preoperative CT or MRI is essential.^13^

Close bone contact and strong internal fixation between adjacent joint fusion surfaces are important factors for successful fusion. Appropriate pressure can bring the fusion surfaces closer together and achieve stability, accelerating the healing process. Factors that influence the choice of internal fixation materials encompass surgeon experience and preference, equipment availability, and time required, among others. For midfoot and hindfoot fixation, screws or plates were commonly used. A research simulating triple arthrodesis on cadavers suggest that there is no statistical difference in fixation strength between screws and staples at the TN, calcaneocuboid, or subtalar joints.^14^ Payette et al. also carried out a trial which showed no statistically significant difference between screws and staples in ultimate load (*p* = 0.4531) and in stiffness (*p* = 0.4636). However, they believed that bone quality has an obvious effect on stability and staples appeared to hold better in softer bone than screws. Additionally, the technical challenges, bone integrity jeopardized, and the time‐consuming nature of screw insertion, the main types of failures were noted including migrating through the cortical bone, fracturing of the dorsal cortex and the midtarsal screws running into the subtalar screw.^15^ Lee et al. investigated the predictor of nonunion following midfoot arthrodesis by comparing the different fixation methods. They revealed that the fixation construct of the compression screw alone was noted to have a significantly higher nonunion rate than staple fixation, compression plate fixation, and compression plate with lag screw fixation. Among 95 patients (99 feet), 5 patients with 8 midfoot joints required revision surgery because of symptomatic nonunion, 11 patients had hardware irritation, and 2 patients developed hardware backing out or breakage without irritation. Diabetes mellitus, lack of adjuvant bone graft, and postoperative non‐anatomical alignment were also risk factors.^16^ Currently, there is no standardized fixation protocol for joint fusion procedures in MWD. It is necessary to make improvements to the existing methods of joint fixation to enhance fusion efficacy.

The challenges encountered during the joint fixation procedure were: (1) Fixation strength was negatively affected by navicular bone avascular necrosis and increased bone fragmentation due to drilling of the screws. (2) Due to specified positioning and spatial limitations, hollow screws cannot be inserted perpendicular to the fusion surface. Also screw fixation might be inadequate to counteract the residual forces arising from the external rotation of the talar head and the adduction forces acting on the navicular, respectively. Poor integration of bone edges can elevate the risk of delayed healing, malunion, and the formation of pseudoarthrosis. There is a comparatively high incidence of nonunion at the TN joint following triple arthrodesis^17^ (3) during postoperative follow‐ups, we found that the fusion may have implant failure with screw or plate breakage^5^ (4) plate fixation requires a larger incision and more extensive dissection of soft tissue, potentially harming adjacent dorsalis pedis arteries and deep peroneal nerves. Moreover, friction between the plate and either the skin or subcutaneous tissue may hinder the healing process of wounds.

### Advantages of the SMA Staple

The SMA staples possess pseudoelasticity (or super‐elasticity) and shape memory characteristics, providing high elasticity, durability, and the ability to undergo reversible shape alterations.^10^ The sensitivity to specific temperature values can be adjusted by altering the ratio of nickel to titanium.^18^ The staples used in this study required pre‐soaking in water at zero degrees to soften them for shaping to the appropriate length.

SMA staples are used for fracture fixation and joint fusion procedures, with a high rate of bone healing.^19^, ^20^, ^21^, ^22^ The markedly high compressive force without the need for bicortical fixation underscores its significant advantage. This kind of staple demonstrated dynamic active compression, where instead of diminishing after loading, the compression actually increased over time.^23^, ^24^ The use of plates requires more hardware for local tissue and demands more time and effort. The use of screws requires accurate placement and struggle to achieve an effective longitudinal compressive force across the fracture site. The inward compressive pressure exerted by the SMA staple, brings the bone fragments together tightly and helps counteract tension caused by muscle pull or changes in joint position. This accelerates bone healing and enables patients to engage in early functional rehabilitation. Additionally, they also offer advantages such as reducing the need for visualization and minimizing irritation to soft tissues.^20^ The reverse sawtooth design also reduces the likelihood of any staples loosening after implantation.

### Clinical Outcomes

This study comprised 8 patients with Stage III MWD and 6 patients with Stage IV MWD, presenting varying degrees of fractures and compressions in the tarsal bones, manifested as adjacent arthritis. In this surgical approach outlined in the study, screws and the SMA staple are placed to fix the TN joint. The screws provide compression, the SMA staples restrict traction and rotation at the joint, preventing screw head penetration through the cortex or screw pullout from cancellous bone, exerting a sustained compression force on joint fusion additionally. Fusion of the midfoot joints enhances tarsal stability, corrects developmental deformities, restores foot alignment, thereby alleviating pain.

Several studies using different fixation methods have shown favorable results with arthrodesis. Harroongroj et al. reported that statistically significant improvement was seen in the radiographic calcaneal pitch angle and VAS scores from the pre‐ to postoperative in 16 patients with MWD who underwent isolated TN arthrodesis.^6^ However, 4 patients required additional locking plates due to instability following intraoperative screw fixation, and 3 patients experienced delayed union complications at 3 months. We believe that if there is a sudden intraoperative decision to add a plate to increase the strength of the fixation, this may affect the precise location of the screws, ultimately affecting the healing outcome. Lu et al. treated 13 feet with MWD with isolated TN arthrodesis using screws and found that TN arthrodesis provided a 76.9% rate.^25^ The union rate was relatively low, suggesting that the fusion may not be strong enough. And two patients underwent implant removal after solid bone union due to hardware prominence. Yuan et al. used a locking steel plate and additional tension screws to fix the TN joint. The AOFAS and VAS scores were significantly improved. The lateral Meary's angle changed from 6.7 to 0.7, the talar‐first metatarsal angle decreased from 15.8 to 7.0, and the calcaneal pitch angle increased from 13.7 to 22.0. They reported 1 patient with fixation delayed after the surgery, and achieved solid fusion at 9 months after removing the implants.^26^ We believed that the fixation with a locking plate and a lag screw could provide good stability, but the procedure is complex and the bone loss is greater than with a SMA staple and screws. Fornaciari et al. took a compression plate as the tension band to provide stability against the counteracting deforming forces.^9^ The AOFAS score increased from 33.0 to 88.3 points postoperatively, and the calcaneal pitch angle was markedly restored. However, plate fixation has disadvantages such as soft tissue irritation, which are mitigated by the SMA staple.

In the study, we found that there was a significant improvement in both the AOFAS score and the VAS score. The AOFAS score increased from 32.2 to 86.6 points and VAS score decreased from 7.4 to 1.2 points. Meanwhile, the lateral Meary's angle changed from 13.5 to 4.1; the calcaneal pitch angle increased from 10.1 to 14.4; the talar‐first metatarsal angle decreased from 11.7 to 4.3. These results illustrate the restoration of the height of the medial longitudinal arch, correction of the midfoot foot alignment, reduction of the subtalar joint and foot deformities that were effectively corrected. All patients in the study recovered and resumed their daily activities. Our study further validates the efficacy of TN arthrodesis in MWD. Besides, we believe this modified fixation method is superior to the exclusive use of screws or the combined use of screws and the plate.

### Limitations and Strengths

This modified fixation method offers the benefits of excellent clinical efficacy in joint fusion, a lower complication rate, and ease of operation. To our knowledge, it is the first study reporting the use of SMA staples in arthrodesis for MWD patients. The results also provide possible directions for arthrodesis. Unfortunately, limitations of this study include its retrospective design, small sample size, absence of a control group, and short postoperative follow‐up. It is hoped that a multicenter, large sample size, prospective, randomized, controlled study of arthrodesis with the assistance of SMA staples will be conducted in the future.

## Conclusions

Using the SMA staple as an adjunct alongside screws in TN arthrodesis surgery to treat MWD provides good clinical outcomes. The study emphasizes the importance of better placed implants to assist bone healing and the efficacy of the SMA staple in clinical applications was further evaluated. Therefore, for surgical interventions targeting MWD, this modified fixation method presents a promising option.

## Ethics Statement

The present study was approved from Zhongshan Hospital of Traditional Chinese Medicine Institutional Review Board review. (NO.2024ZSZY‐LLK‐374).

## Conflict of Interest Statement

We declare that we have no competing interests.

## Author Contributions

LZ‐Y designed the study. LZ‐Y, and WB‐H collected the data. LZ‐Y and TY‐H wrote the manuscript. ZY‐Z, LX‐Y and LY‐Q revised the manuscript. LZ‐Y and ZC‐X approved the final manuscript for submission.

## References

[1] Ahmed ASAA , Kandil MI , Tabl EA , Elgazzar AS . Müller‐Weiss disease: atopical review. Foot Ankle Int. 2019;40(12):1447–1457. 10.1177/1071100719877000 31538823

[2] Maceira E , Rochera R . Müller‐Weiss disease: clinical and biomechanical features. Foot Ankle Clin. 2004;9(1):105–125. 10.1016/S1083-7515(03)00153-0 15062217

[3] Wong‐Chung J , Walls A , Lynch‐Wong M , Cassidy R , McKenna R , Wilson A , et al. Towards understanding Müller‐Weiss disease from an analysis of 95 cases. Foot Ankle Surg. 2023;29(5):401–411. 10.1016/j.fas.2023.05.004 37225610

[4] Harnroongroj T , Tharmviboonsri T , Chuckpaiwong B . Müller‐Weiss disease: the descriptive factors of failure conservative treatment. Foot Ankle Int. 2021;42(8):1022–1030. 10.1177/10711007211002826 33843318

[5] Lee TY , Wu CC , Yang KC , Yeh KT , Chen IH , Wang CC . Midterm outcomes of midfoot and hindfoot arthrodesis with strut allograft for Müller‐Weiss disease. BMC Musculoskelet Disord. 2022;23(1):715. 10.1186/s12891-022-05629-7 35897013 PMC9327191

[6] Harnroongroj T , Chuckpaiwong B . Müller‐Weiss disease: three‐ to eight‐year follow‐up outcomes of isolated Talonavicular arthrodesis. J Foot Ankle Surg. 2018;57(5):1014–1019. 10.1053/j.jfas.2018.01.008 29804921

[7] Bai W , Li Y , Shen G , Zhang H , Li X , Zhu Y . Talonavicular‐cuneiform arthrodesis for the treatment of Müller‐Weiss: mid‐term results of 15 cases after 5 years. BMC Musculoskelet Disord. 2023;24(1):178. 10.1186/s12891-023-06293-1 36894915 PMC9999486

[8] Lu L , Liu B , Zeng J , Chen W , Hu F , Ma Q , et al. Efficacy of triple and Talonavicular arthrodesis for the treatment of III‐V Müller‐Weiss disease. Tohoku J Exp Med. 2022;258(2):97–102. 10.1620/tjem.2022.J062 35896365

[9] Fornaciari P , Gilgen A , Zwicky L , Horn Lang T , Hintermann B . Isolated Talonavicular fusion with tension band for Müller‐Weiss syndrome. Foot Ankle Int. 2014;35(12):1316–1322. 10.1177/1071100714548197 25139862

[10] Wu JC , Mills A , Grant KD , Wiater PJ . Fracture fixation using shape‐memory (Ninitol) Staples. Orthop Clin North Am. 2019;50(3):367–374. 10.1016/j.ocl.2019.02.002 31084839

[11] Li SY , Myerson MS , Monteagudo M , Maceira E . Efficacy of calcaneus osteotomy for treatment of symptomatic Müller‐Weiss disease. Foot Ankle Int. 2017;38(3):261–269. 10.1177/1071100716677741 27838679

[12] Cao HH , Lu WZ , Tang KL . Isolated talonavicular arthrodesis and talonavicular‐cuneiform arthrodesis for the Müller‐Weiss disease. J Orthop Surg. 2017;12(1):83. 10.1186/s13018-017-0581-4 PMC5460349 28583192

[13] Liu W , Chen Y , Zeng G , Yang T , Ma M , Song W . Individual surgical treatment of stage IV Müller‐Weiss disease according to CT/MRI examination: aretrospective study of 12 cases. Front Surg. 2022;9:694597. 10.3389/fsurg.2022.694597 35372477 PMC8968067

[14] Meyer MS , Alvarez BE . Triple arthrodesis: a biomechanical evaluation of screw versus staple fixation. Foot Ankle Int. 1996;17:764–767. 10.1177/107110079601701209 8973900

[15] Payette CR , Sage RA , Gonzalez JV , Sartori M , Patwardhan A , Vrbos L . Triple arthrodesis stabilization: a quantitative analysis of screw versus staple fixation in fresh cadaveric matched‐pair specimens. J Foot Ankle Surg. 1998;37(6):472–480. 10.1016/s1067-2516(98)80024-0 9879042

[16] Lee W , Prat D , Wapner KL , Farber DC , Chao W . Comparison of 4 different fixation strategies for Midfoot arthrodesis: aretrospective comparative study. Foot Ankle Spec. 2024;17(2):98–108. 10.1177/19386400211032482 34340573

[17] Cates NK , Mayer A , Tenley J , Wynes J , Tefera E , Steinberg JS , et al. Double versus triple arthrodesis fusion rates: asystematic review. J Foot Ankle Surg. 2022;61(4):907–913. 10.1053/j.jfas.2022.01.012 35221217

[18] Mahtabi MJ , Shamsaei N , Mitchell MR . Fatigue of Nitinol: the state‐of‐the‐art and ongoing challenges. J Mech Behav Biomed Mater. 2015;50:228–254. 10.1016/j.jmbbm.2015.06.010 26160028

[19] Rocchi L , Merendi G , Cazzato G , Caviglia D , Donsante S , Tulli A , et al. Scaphoid waist fractures fixation with staple. Retrospective study of a not widespread procedure. Injury. 2020;51:S2–S8. 10.1016/j.injury.2019.12.020 31902574

[20] Posey SL , Gaston RG . Staple technology for fracture fixation and joint arthrodesis. Hand Clin. 2023;39(4):505–513. 10.1016/j.hcl.2023.05.010 37827603

[21] Choudhary RK , Theruvil B , Taylor GR . First metatarsophalangeal joint arthrodesis: a new technique of internal fixation by using memory compression staples. J Foot Ankle Surg. 2004;43(5):312–317. 10.1053/j.jfas.2004.07.003 15480407

[22] Richards T , Newington D . The results of carpometacarpal joint arthrodesis of the osteoarthritic thumb in younger patients using memory Staples. J Hand Microsurg. 2022;14(3):266–267. 10.1055/s-0040-1716613 36016631 PMC9398576

[23] Hoon QJ , Pelletier MH , Christou C , Johnson KA , Walsh WR . Biomechanical evaluation of shape‐memory alloy staples for internal fixation—an in vitro study. J Exp Orthop. 2016;3(1):19. 10.1186/s40634-016-0055-3 27578288 PMC5005248

[24] McKnight RR , Lee SK , Gaston RG . Biomechanical properties of Nitinol Staples: effects of Troughing, effective leg length, and 2‐staple constructs. J Hand Surg. 2019;44(6):520.e1–520.e9. 10.1016/j.jhsa.2018.08.017 30344022

[25] Lu CK , Fu YC , Cheng YM , Huang PJ . Isolated talonavicular arthrodesis for Müller‐Weiss disease. Kaohsiung J Med Sci. 2014;30(9):471–476. 10.1016/j.kjms.2014.05.001 25224771 PMC11916762

[26] Yuan C , Wang C , Zhang C , Huang J , Wang X , Ma X . Derotation of the talus and arthrodesis treatment of stages II‐V Müller‐Weiss disease: midterm results of 36 cases. Foot Ankle Int. 2019;40(5):506–514. 10.1177/1071100719829457 30776926

